# The positive impact of mindfulness interventions on the explicit and implicit affective attitudes toward vegetarian foods

**DOI:** 10.3389/fpsyg.2023.1158410

**Published:** 2023-10-04

**Authors:** Annica Winkelmair, Petra Jansen

**Affiliations:** Faculty of Human Sciences, University of Regensburg, Regensburg, Germany

**Keywords:** mindfulness, attitudes, priming paradigm, vegetarian food, sustainable food consumption, sustainability, behavioral change, intervention study

## Abstract

**Objectives:**

The main goal of our intervention study was to investigate whether two conceptually different mindfulness interventions positively impacted the explicit and implicit affective evaluations of vegetarian foods. We included possible mediating variables (e.g., wellbeing) and related our results to the stage model of self-regulated behavioral change (SSBC).

**Methods:**

We implemented a compassion and caring-based mental training (*N* = 31) and an adapted MBSR course (*N* = 34) as mindfulness interventions, and a stress-reduction course (*N* = 26) as the active control group. The curriculums consisted of 12 weekly group sessions á 75 min. All participants were tested pre- and post-intervention and 3 months after the last intervention session, answered questionnaires (mindfulness, compassion, wellbeing, items of the SSBC) and completed an explicit affective evaluation task and an affective priming task.

**Results:**

There was an improvement in the explicit attitudes toward vegetarian foods regardless of the intervention group. In the SSBC, we found a link between the explicit attitudes toward vegetarian foods and the indicated stage in the model. Multiple regression analysis revealed social and personal norms and a vegetarian/vegan diet as the only significant predictors for goal intention in the SSBC.

**Conclusion:**

The results of our study suggest that both conceptually different mindfulness interventions, as well as a stress-reduction program, have a positive impact on explicit affective attitudes toward vegetarian foods. We highlight the meaning of inner dimensions and transformation for change processes for a more sustainable diet and the role of social and personal norms.

## 1. Introduction

The Intergovernmental Panel on Climate Change (IPCC) report states that “it is unequivocal that human influence has warmed the atmosphere, ocean, and land” (Allan et al., 2021, p. 4). A change of human behavior in a more sustainable direction thus seems inevitable. There is also an understanding that regarding sustainability, we need to focus more on the inner worlds, like emotions, thoughts, and beliefs, instead of addressing the climate crisis solely on collective or technological levels (Ives et al., 2020). Current research highlights the importance of the inner transformation that relates, for example, to values as a dimension of sustainability transformations (Woiwode et al., 2021). A noteworthy contribution to pro-environmental behavior at the individual level involves adopting a sustainable diet. The production and consumption of food can account for 19–29% of global anthropogenic greenhouse gas (GHG) emissions (Vermeulen et al., 2012). Hence, the food sector represents a decisive area for action, and the decision for a sustainable form of nutrition significantly contributes to personal sustainability. There are different ways of following a sustainable diet, like the preference for organic, regional, or seasonal foods but also the reduction of animal products such as meat (Von Koerber et al., 2017). However, according to the United Nations, a global development towards a plant-based diet can make a significant contribution to saving the world from the greatest damage of climate change (Alvaro, 2017). Thus, a vegetarian or even vegan diet is considered a promising and beneficial form of sustainable nutrition. Meanwhile, it is widely acknowledged that deficiencies in protein, a significant macronutrient in meat-based diets, do not necessarily manifest in vegetarian or vegan diets. Moreover, health benefits seem to arise from plant-based protein sources (Ewy et al., 2022). There are various positive effects of vegetarian nutrition—besides its lower environmental impact—such as better physical health, more positive feelings for moral reasons, and overall higher quality of life (Hargreaves et al., 2021). However, only 7% of the German population reported eating vegetarian (Statista, 2020). Accordingly, there is a high interest in promoting vegetarianism and, thus, a promising way to eat sustainably. Current research acknowledges the concept of mindfulness as a mechanism to foster sustainable consumption behavior and lifestyle (e.g., Ericson et al., 2014; Fischer et al., 2017; Geiger et al., 2020). Since most of the previous studies showed only small effects and were cross-sectional (Geiger et al., 2019), our study adds to this lack of research by investigating the potential impact of different mindfulness interventions on the affective attitudes toward vegetarian foods in a randomized controlled longitudinal design.

Mindfulness can be described as “the awareness that emerges through paying attention on purpose, in the present moment, and nonjudgmentally to the unfolding of experience moment by moment” (Kabat-Zinn, 2003, p. 145). It can be regarded in several ways: as a state that can be achieved through meditation, a dispositional trait, a type of meditation practice, or an intervention (Vago and Silbersweig, 2012). Mindfulness as a trait can be increased by regular meditation practice and thereby caused neuroplastic changes (Hölzel et al., 2011). Different forms of meditation can be used as elements of mindfulness interventions, such as attentional or constructive meditation practices, according to the classification scheme of Dahl et al. (2015). Attentional practices aim to train processes related to the regulation of attention and strengthen the cognitive function for being aware of the processes of thinking, feeling, and perceiving. It can be differentiated between focused-attention and open-monitoring practices. Focused-attention practices involve a narrowing of the attentional scope and concentration on one single object, such as breath counting (Lutz et al., 2008). Open-monitoring practices involve an expansion of the attentional scope and, thus, a flow of perceptions, thoughts, and awareness. A well-established and evidence-based example of open-monitoring meditation practice is the Mindfulness-based stress-reduction (MBSR) program. In this intensive mindfulness training, the individual learns to observe experiences instead of being wholly immersed (Kabat-Zinn, 1982). It includes formal mindfulness practices to increase attentional control and the non-judgmental attitudinal aspects of mindfulness. Constructive meditation practices strengthen psychological patterns that foster wellbeing by replacing maladaptive self-schemes with more adaptive self-understandings.

In contrast to the attentional family, meditation forms of the constructive family involve an active and systematic change in the cognitive and affective contents instead of monitoring and simply observing the present thoughts and emotions (Dahl et al., 2015). A widely used form of meditation within the constructive family of meditation forms is loving-kindness meditation (LKM; Lippelt et al., 2014). LKM focuses on developing love for oneself, a beloved person, a stranger, and a person one does not like. This style of practice can enhance mindfulness as well as the awareness of the own environment. With its focus on warm-heartedness, it can also increase positive emotions, emotional wellbeing (Fredrickson et al., 2017), a sense of connectedness toward others (Hutcherson et al., 2008), and compassion (Luberto et al., 2018) and impact prosocial behavior (Böckler et al., 2018).

The concept of mindfulness is discussed as a potential related factor for sustainability in sustainable consumption research. A core quality of mindfulness is the ability to disengage from an automated thought-processing mode (Rosenberg, 2004) and enable more conscious choices by the disruption of routines. Besides that, Fischer et al. (2017) identified at least three other mechanisms of trait mindfulness for sustainable consumption: congruence of attitude and behavior, non-material values and wellbeing, and prosocial behavior. However, in addition to its qualities of awareness, the gentle emotional quality of mindfulness (Kabat-Zinn, 2003) that can be experienced through exercises of the constructive family such as LKM, can also be seen in relation to sustainable consumption as it fosters pro-environmental tendencies (Pfattheicher et al., 2016) and sustainable decision making (Engel et al., 2020). As mentioned above, the practice of LKM strengthens prosociality (Böckler et al., 2018) and increases feelings of social connection (Hutcherson et al., 2008). These attributes, in turn, have been observed to be linked with sustainable behavior (de Groot and Thøgersen, 2012). In the present study, we focus on the potential role of two conceptual different mindfulness interventions—one rather cognitive, awareness focused and one rather compassion oriented—for vegetarian food consumption.

The connection between mindfulness facets and sustainable nutrition has been investigated in a few studies so far. For example, Jacob et al. (2009) found a significant link between sustainable food practice and the frequency of mindfulness meditation. Hunecke and Richter (2019) investigated the relation of five dispositional mindfulness facets (observing, describing, acting with awareness, nonjudging of inner experience, nonreactivity to inner experience) with the following constructs: construction of meaning in life, sustainability-related meaning, personal ecological norm, and sustainable food consumption. Their study revealed a direct relationship between the mindfulness facet acting with awareness and self-reported sustainable food consumption. An enhancement in this dimension of mindfulness might thus support the choice of sustainable food. However, this direct relation was only observed for sustainable food choices but not for a vegetarian lifestyle which might be more influenced by moral norms like animal welfare and ecological norms. The follow-up study of Richter and Hunecke (2020) provides a theoretical approach to how different dimensions of mindfulness and the change process of human behaviors towards organic food consumption are linked. Their model is based on the stage model of self-regulated behavioral change (SSBC) by Bamberg (2013), which incorporates variables of the theory of planned behavior (Ajzen, 1991), the norm-activation model (Schwartz, 1977), and stages of behavioral action adapted from the mindset theory of action phases by Gollwitzer (1990). In the context of nutrition, the SSBC of Bamberg (2013) has been applied to beef consumption by Klöckner (2017). This investigation revealed attitudes as the main determinants for the choice of an alternative behavior, e.g., the substitution of beef with other meats or seafood, or vegetarian meals, and emphasizes the role of social norms and the awareness of negative consequences of behaviors through personal norms for the goal intention of reducing beef consumption. In the framework of Richter and Hunecke (2020), an adapted and reduced version of the SSBC by Bamberg (2013) was used, comprising different types of intentions (goal intention, behavior intention, implementation intention) and a fixed sequence of stages toward behavioral change (pre-decision, pre-action, action, and post-action stages). They include stage-specific variables that influence intentions and behavior such as social norms, personal norms, attitudes, perceived behavior control, and different forms of self-efficacy. Their cross-sectional online study revealed a significant relation between the mindfulness facet observing and goal intention and an indirect effect on goal intention towards organic food consumption, which was mediated by social and personal norms, and explicit attitudes. The predictive value of personal and social norms, attitudes, and perceived behavior control thus must be considered. Siebertz et al. (2022) applied the adapted SSBC by Richter and Hunecke (2020) in the context of vegetarian and vegan food consumption and dispositional mindfulness. Their results showed that the mindfulness facet observing correlated with the explicit attitudes and goal intention and that personal norms mediated the link between observing and goal intention. However, in the SSBC, only explicit attitudes are considered. But besides these controlled-conscious attitudinal aspects there is also an implicit, rather uncontrolled-unconscious dimension of human attitudes as dual-process models propose (Cameron et al., 2012). Combining both explicit and implicit measurements can help reveal underlying attitudes and explain the willingness for behavioral changes. By including implicit attitudes in the model, the SSBC could benefit from capturing not only self-reports but also aspects that are rather unavailable to consciousness.

Conscious attitudes can be assessed through explicit measurements, e.g., direct questions, whereas subconscious attitudes require implicit measurements, like the affective priming task focusing on the affective component of implicit evaluations. In this paradigm, the response latency on a target stimulus after the presentation of a prime stimulus is measured (De Houwer et al., 2009). However, research has proved that a person’s explicit and implicit attitudes are not always related (Cameron et al., 2012). There is a growing awareness that individual consumption decisions are also influenced by an automatic, unconscious component (Panzone et al., 2016). Especially in the field of sustainability, considering both dimensions of attitudes might be crucial as previous research revealed a low congruence between explicit and implicit sustainability orientations (Steiner et al., 2018). Consistent with this finding, Jansen et al. (2021) discovered a more positive explicit attitude towards e-mobility compared to gasoline cars, while no higher affective implicit rating could be observed. In the context of nutrition, previous research showed that explicit attitudes toward vegetarian and vegan foods depend on the preferred diet: Omnivores rated pictures of meat-based food as more positive, and non-omnivores vegetarian and vegan food. Nevertheless, all participants rated non-omnivorous food implicitly more positively (Siebertz et al., 2022). In addition, another study using actual supermarket shopping data could demonstrate that explicit and implicit attitudes influence consumer decisions differently in specific food categories (Panzone et al., 2016). These findings suggest that research in sustainability and sustainable behavior could yield important insights by exploring both explicit and implicit attitudes and their (in)congruence (Steiner et al., 2018).

Mindfulness interventions might help to reconcile the unconscious and conscious aspects of attitudes. Previous research suggests that due to focusing on the current situation, mindfulness meditation can reduce the impact of past experiences on the present moment and therefore lead to reduced activation of automatic associations (Lueke and Gibson, 2015). One central idea of mindfulness is that it increases awareness of impulses, and while accepting these events, a person can prevent automatically acting on them. Thus, mindfulness has the potential to enhance controlled processes while regulating automatic processes (Karremans and Papies, 2017).

The main goal of this study was to investigate whether a cognitive oriented mindfulness intervention and a compassion and caring-based intervention that involves meditation forms of the constructive family positively impact the explicit and implicit affective evaluations of vegetarian foods as a form of sustainable nutrition. We implemented two conceptual different mindfulness training programs: an adapted MBSR training and a compassion and caring-based mental training including LKM and affect dyads. As an active control group, a third group received stress-reduction training. Since previous research emphasizes the significance of mediating factors in the relationship between mindfulness and sustainable consumption, we included possible influencing variables such as wellbeing and compassion. Also, we applied the modified version of the SSBC of Richter and Hunecke (2020) and investigated the role of explicit and implicit affective attitudes for goal intention and their link to stage affiliation towards a vegetarian diet. Our hypotheses are as follows:

*H1*: First, since previous research showed a correlation between mindfulness and affective attitudes (e.g., Jansen et al., 2021), we assume that both the adapted MBSR and the compassion and caring-based intervention groups improve the explicit and implicit affective attitudes toward vegetarian foods compared to the active control group. If the improvement is due to a change in the daily awareness of impulses (Karremans and Papies, 2017; Hunecke and Richter, 2019), it should be higher after the attention-focused meditation training (adapted MBSR) compared to the compassion and caring-based mental training. On the other hand, if the improvement is due to a change in the feeling of connectedness to others (Hutcherson et al., 2008) and a higher prosocial behavior (Böckler et al., 2018), it should be the other way around.

*H2*: According to Richter and Hunecke (2020), the behavioral change toward sustainable food consumption follows a fixed sequence of stages. We, therefore, hypothesize that a higher stage in the SSBC is linked to a more positive attitude toward vegetarian food.

*H3*: In the SSBC of Richter and Hunecke (2020), not only (explicit) attitudes and the facets of mindfulness but also personal and social norms are related to goal intention in the pre-decision stage. For this, we would like to investigate if mindfulness trainings predict together with the attitudes toward vegetarian foods, social and personal norms, a vegetarian diet, the different mindfulness facets, compassion, and wellbeing the goal intention for a vegetarian diet.

*H4*: In line with the results of Steiner et al. (2018), Jansen et al. (2021), and Siebertz et al. (2022), we expect only a marginal correlation between the explicit and implicit affective ratings in the pre-test, if any. However, since mindfulness might be a factor that could lead to reduced activation of automatic associations (Lueke and Gibson, 2015), we hypothesize a stronger correlation between the explicit and implicit affective attitudes after the interventions.

## 2. Materials and methods

### 2.1. Participants

Using G*power (Faul et al., 2007), power analysis for the repeated measurements ANOVA within-between interaction (within factor: three time points of measurement, between factor: three groups) of our central hypothesis 1 was performed. Consequently, with a small effect size of *f* = 0.15, a power of 1–ß = 0.80, and a standard alpha probability of 0.05, we aimed for a total sample size of *N* = 93. Participants were recruited through the institute of sports science newsletter at the University of Regensburg and student groups on social media. Students of sports science received course credit for their participation. However, there was no academic connection between the investigator and the subjects to control for possible social desirability effects. The study was conducted in line with the ethical guidelines of the Helsinki Declaration and approved by the ethics board of the University of Regensburg (Reference number: 20-1740-101). It was preregistered prior to data collection at OSF.^1^ All participants were informed and gave their written consent. We collected the data of 119 participants pre-intervention, 98 post-intervention, and 94 at the time of follow-up measurement three months after the intervention. Six participants left the study between the pre-test and follow-up in the compassion and caring-based mental training group, four in the adapted MBSR group, and seven in the active control group. Eight subjects left the study before the intervention groups were assigned. The primary reasons for dropout during the 12-week intervention groups were the change of study program, prolonged illness, and lack of time for attending the weekly group sessions. In addition, three more participants had to be excluded due to more than 50% incorrect responses in the affective priming task, resulting in a final sample size of 91 participants (compassion and caring-based mental training group: *N* = 31, adapted MBSR group: *N* = 34, active control group: *N* = 26) consisting of 54 women and 37 men (*M* age = 22.44, *SD* = 2.39).

**Figure 1:**
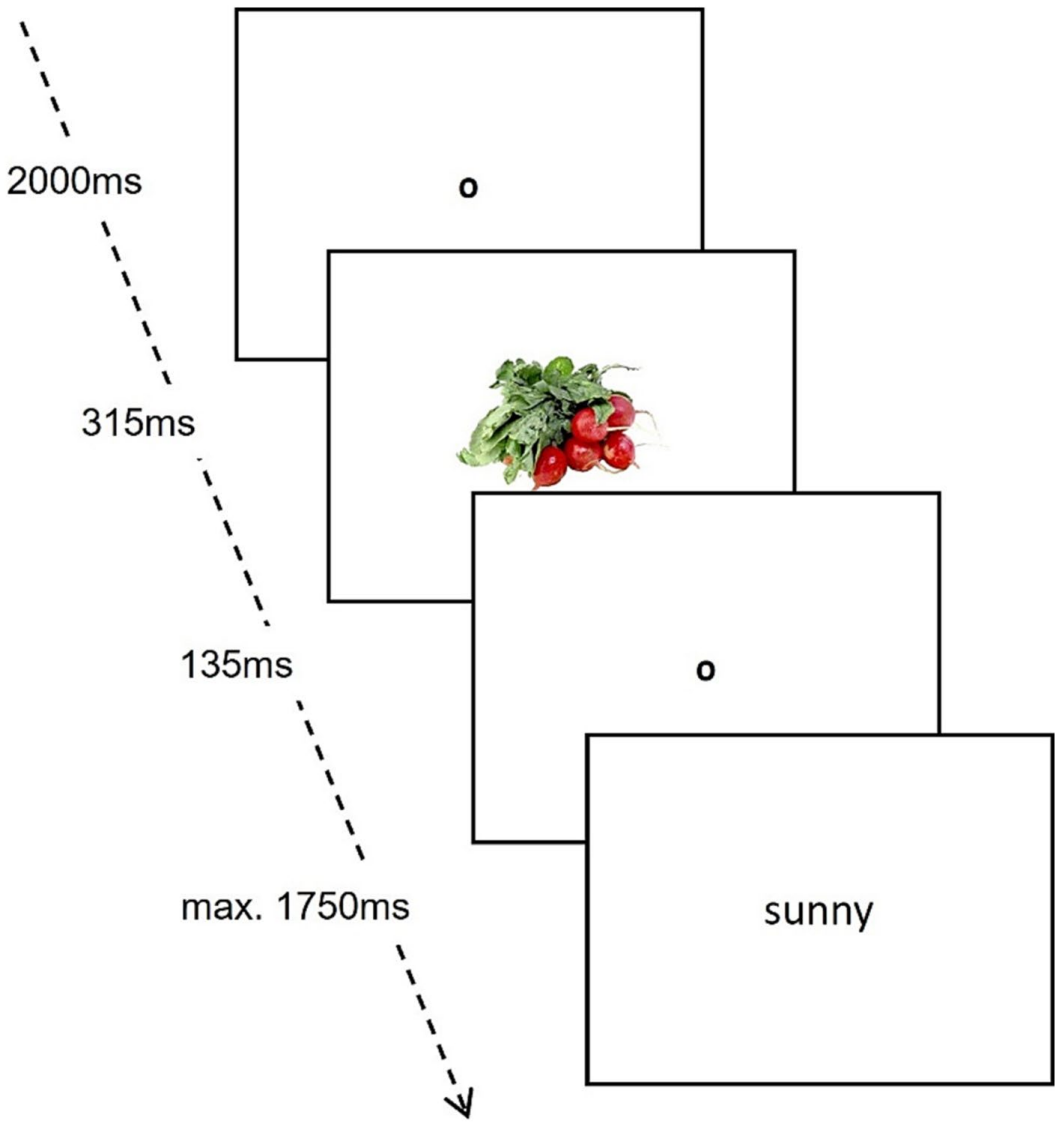
Procedure of the implicit affective priming task.

### 2.2. Procedure and design

#### 2.2.1. Intervention

We implemented two conceptually different mindfulness curriculums as interventions and an active control group. All three programs consisted of 12 weekly group sessions á 75 min and weekly homework assignments. To keep group sizes as small as possible, we scheduled two groups at different times for each program, resulting in subgroup sizes of between 17 and 21 participants. Experienced trainers with specific mindfulness education taught all groups. No sustainability-related content was discussed or implemented in any of the groups.

##### 2.2.1.1. Compassion and caring-based mental training

The core exercises of this curriculum were LKM (Salzberg, 2004) and affected dyads. In LKM, the participants are introduced to a way to offer love to themselves, other people, or animals. People are asked to mentally repeat phrases like “May I/you be happy,” “May I/you be healthy,” “May I/you be safe,” and “May I/you live with ease” (Trautwein et al., 2020). Furthermore, affect dyad situations were applied where partners sit face to face, and one partner contemplates a situation to a specific theme of the main topics. The other partner listens attentively without giving any verbal or non-verbal feedback. Each session included a talk about a specific topic. The main subjects of this course were breath, handling difficult emotions, the four Buddhist Brahmaviharas, prosociality, compassion, appreciative joy, equanimity, forgiveness, gratitude, and self-compassion. All sessions followed a fixed sequence of quiet time, check-in, talk, meditation, exercise, homework, and check-out.

##### 2.2.1.2. Adapted MBSR training

The adapted MBSR training was taught in line with the original course of Kabat-Zinn (1990). The well-established curriculum includes the following elements of meditation: body scan, mindful eating, emphasizing the breath, and sitting and walking meditation training. Every session contained a talk about specific topics such as different forms of meditation and problems that may arise with them, different levels of sensation during meditation, coping with complex thoughts and feelings, being mindful of the body, and attentional control. The focus of this mindfulness group was on the rather cognitive elements of mindfulness and its qualities of awareness. In line with the compassion and caring-based mental training curriculum, all sessions followed the same fixed sequence of quiet time, check-in, talk, meditation, exercise, homework, and check-out.

##### 2.2.1.3. Active control group

We implemented a stress-reduction program as an active control group. This course taught progressive muscle relaxation (PMR), as it had already been used in mindfulness research (Gao et al., 2018). The curriculum was taught in line with the manual by Hofmann (2020) and included training in lying, sitting, and imagination practice. There was no overlap in content between the active control group and the two mindfulness interventions, and strict consideration was given to avoid any mindfulness-related exercises in this stress-reduction program.

#### 2.2.2. Experimental pre/post/follow-up design

We applied a three (group: compassion and caring-based mental training, adapted MBSR, active control group) x three (time: pre-test, post-test, follow-up) design. Participants were randomly assigned to one of the three groups. They were unaware of their group assignment and were instructed not to interact with other study participants of the other two groups about the content of the group sessions. Participants had to attend at least nine weekly meetings (this differs from our preregistered exclusion criterium of the maximum of two missing sessions, but we decided to mitigate this criterium due to the current Covid-19 situation). There were three time points of measurement: (1) within two weeks before starting of the intervention groups (pre-test), (2) within two weeks after the last group sessions (post-test), and (3) around three months after the last group sessions (follow-up). We included a follow-up measurement to assess longer-term changes beyond the intervention sessions. We chose a three-month period as this period corresponds to the semester break and thus the post-tests were conducted at the end of the semester and the follow-up tests at the beginning of the new semester. The tests lasted about 20 min each and included questionnaires presented *via* the software Sosci Survey (Leiner, 2019) and two tasks for measuring explicit and implicit attitudes. Both tasks were programmed in OpenSesame (Mathôt et al., 2012).

### 2.3. Measures

We reported McDonald’s Omega as an internal consistency index and used the pre-test data (*N* = 119) for its calculation.

#### 2.3.1. Demographic data

Questions concerning age, gender, education state, mother tongue, regular occupation, and family status were asked. Furthermore, the frequency and average duration in minutes of practicing yoga and meditation were assessed during the pre-test. In addition, questions regarding personal diet were asked: we registered eating habits (vegan, vegetarian, and omnivorous) and the personal importance of the own nutrition at all three measurement times to record change over the intervention. Last, we measured engagement with the content of the groups beyond the weekly sessions in the form of home assignments at the post-test and engagement with the material after the last group session at the follow-up measurement in weekly minutes.

#### 2.3.2. Goal intention, stage affiliation, social and personal norms

For the implementation of the SSBC, goal intention, social and personal norms, according to Richter and Hunecke (2020), were assessed at all three measurement times. Two items measured the goal intention to eat more vegetarian meals (*r* = 0.84, *p* < 0.001) on a five-point Likert scale from 1 (“does not apply”) to 5 (“fully applies”). Also, social norm—the attitude of people considered personally necessary toward vegetarian meals—was measured by two items (*r* = 0.67, *p* < 0.001) and personal norm, in the sense of the own values toward vegetarian meals, by four items (*ω* = 0.80). The items of both norms had to be answered on a five-point Likert scale from 1 (“does not apply”) to 5 (“fully applies”). We calculated the mean value for goal intention and social and personal norms for the corresponding items. Furthermore, stage affiliation was determined by one single-choice item with four options, one option for each stage.

#### 2.3.3. Mindfulness

Aspects related to mindfulness were measured by the German Five Facets Mindfulness Questionnaire (FFMQ; Baer et al., 2008; Michalak et al., 2016). The FFMQ comprises 39 items on five dimensions: observing (*ω* = 0.77; e.g., “I notice the smells and aromas of things.”), describing (*ω* = 0.93; e.g., “I am good at finding words to describe my feelings.”), acting with awareness (*ω* = 0.86; e.g., “I find myself doing things without paying attention.” [R]), nonjudging of inner experience (*ω* = 0.93; e.g., “I think some of my emotions are bad or inappropriate, and I should not feel them.” [R]) and nonreactivity to inner experience (*ω* = 0.84; e.g., “I perceive my feelings and emotions without having to react to them.”). All items had to be rated on a five-point Likert scale from 1 (“applies very rarely”) to 5 (“applies very often”), and the mean values for all five scales were composed.

#### 2.3.4. Compassion

Compassion was assessed using the Compassion Scale (CS; Pommier et al., 2020). It comprises 16 items on four subscales: kindness (e.g., “I like to be there for others in times of difficulty.”), common humanity (e.g., “Everyone feels down sometimes, it is part of being human.”), mindfulness (e.g., “I pay careful attention when other people talk to me.”), and (inverted) indifference, separation, and disengagement (e.g., “I do not concern myself with other people’s problems.”, “I cannot really connect with other people when they are suffering.”, “I do not think much about the concerns of others.”). In the present study, a translated German version applied by Siebertz et al. (2022) was used. The participants stated how often they feel or behave in a specific way on a five-point Likert scale from 1 (“almost never”) to 5 (“almost always”). It should be noted that the CS subscale mindfulness differs conceptually from mindfulness as it was assessed by the FFMQ since its items concern interpersonal relationships in contrast to the latter. We computed the mean value of all 16 items (*ω* = 0.72).

#### 2.3.5. Wellbeing

Wellbeing was measured by the Brief Inventory of Thriving (BIT; Su et al., 2014; Hausler et al., 2017). The BIT consists of ten items (e.g., “I am optimistic about my future”) that had to be evaluated on a five-point Likert scale from 1 (“strongly disagree”) to 5 (“strongly agree”). The mean value over all items was composed (ω = 0.80).

#### 2.3.6. Explicit affective attitudes

We used an explicit affective evaluation task presenting five pictures of vegetarian foods and five meat dishes in random order. The pictures were derived from the food-pics extended image dataset (Blechert et al., 2019) and were matched in terms of familiarity, arousal, and valence using the ratings provided by the database. This same set of pictures has been used in the study of Siebertz et al. (2022). Also in line with the study design of Siebertz et al. (2022), we asked the following question for each picture: “How much do you like the food in the picture?.” They had to answer on a seven-point Likert scale from 1 (“not at all”) to 7 (“very much”) within 5 s to assess their spontaneous reaction. The mean scores for the explicit rating of vegetarian and meat-based foods were calculated.

#### 2.3.7. Implicit affective attitudes

An affective priming paradigm (Fazio et al., 1995; Hutcherson et al., 2008) using the same pictures of the explicit evaluation task was applied. We implemented a short practice trial with four other non-food-related pictures before the central part of the task started. First, an initial fixation point was shown for 2000 ms in the center of the screen. After this, a picture of either a vegetarian or a meat dish appeared briefly for 315 ms. After another fixation point for 135 ms, a word picked randomly from a pool of four positive and four negative words retrieved from the Berlin Affective Word List (BAWL-R; Võ et al., 2009) was shown. The participants had to decide whether the shown word was positive or negative *via* the arrow keys and react as quickly as possible since the word disappeared after 1750 ms, see Figure 1. Each picture was combined with each word, resulting in 80 trials. If participants skipped a trial by answering too slowly, the trial with its respective picture-word combination was repeated at the end of the task. On average, over the 80 trials, *M* = 4.69 (*SD* = 9.15) in the pre-test, *M* = 2.66 (*SD* = 5.07) in the post-test, and *M* = 2.16 (*SD* = 2.43) in the follow-up, had to be imputed due to incorrect or too fast (below 100 ms) responses. After checking visually that empty values were missing at random, they were imputed by multiple imputation algorithms and pooling means. Subsequently, reaction times when categorizing picture-primed positive words were subtracted from reaction times when categorizing picture-primed negative words, separately for both picture categories and averaged, respectively. Thus, a higher difference score reflected a more positive attitude.

#### 2.3.8. Personal evaluation of sustainability

The participants had to indicate on a seven-point Likert scale from 1 (“not at all”) to 7 (“very much”) how sustainable they evaluated the vegetarian and meat dishes shown in the pictures used in the explicit affective evaluation task and the affective priming paradigm. We separately composed the mean value for the vegetarian (*ω* = 0.68) and the meat-based (*ω* = 0.82) food pictures.

### 2.4. Statistical analysis

A three (time: pre-test, post-test, follow-up) x three (group: compassion and caring-based mental training, adapted MBSR, active control group) ANOVA with repeated measurements was conducted to find out if the intervention groups had an impact on the explicit and implicit affective attitudes toward vegetarian foods (hypothesis 1). To analyze if stage affiliation affects the explicit and implicit affective attitudes toward vegetarian foods (hypothesis 2), two one-way ANOVAs with stage affiliation as an independent variable were performed for all three measurements. Multiple linear regression analyses for the post-test and follow-up measurement were calculated for the dependent variable goal intention and the following predictors (hypothesis 3): group (compassion and caring-based mental training, adapted MBSR, active control group), explicit and implicit affective attitudes toward vegetarian foods, social norm, personal norm, vegetarian/vegan diet, the aspects of mindfulness (observing, nonreactivity, acting with awareness, nonjudging, describing), compassion and wellbeing. To account for multicollinearity, variance inflation factors (< 2.05) and tolerance (> 0.48) were considered and regarded as appropriate (O’Brien, 2007). The correlations between the explicit and implicit affective attitudes were calculated for all three measurement times (pre-test, post-test, and follow-up) and both food categories separately (hypothesis 4). We conducted a matched-pairs *t*-test to test whether there was a difference between the explicit rating of vegetarian and meat-based foods. Likewise, to analyze if there was a difference between the implicit affective ratings of meat and vegetarian foods, another matched-pairs *t*-test was performed for the reaction time difference score between negative and positive words. Last, we performed another matched-pairs *t*-test to test whether there was a difference in the personal evaluation of sustainability between the two categories of vegetarian and meat-based foods. Exploratorily, a possible change in mindfulness, compassion, and wellbeing due to the intervention was examined. The Greenhouse–Geisser adjustment was used for relevant results to correct for violations of sphericity. Analyses were performed using IBM Statistics SPSS 28.

## 3. Results

### 3.1. Demographic data

Age, education state, frequency of practicing meditation and yoga (at pre-test), as well as done home assignments (at post-test), and engagement with the contents of the group after the last session (at follow-up) are shown separately for each the three intervention groups in Table 1. The age of the participants differed between the three groups, *χ*^2^(2) = 9.72, *p* = 0.008, as well as the number of attended group sessions (compassion and caring-based mental training group: *M* = 10.29, *SD* = 0.74, adapted MBSR group: *M* = 10.44, *SD* = 0.89, active control group: *M* = 9.85, *SD* = 0.63; *χ*^2^(2) = 8.28, *p* = 0.016), but with no difference between the two mindfulness intervention groups. In addition, eating habits (vegetarian/vegan, omnivorous), the importance of their nutrition, and the three highest-rated reasons if a vegetarian/vegan diet was chosen are presented in Table 2 over all three points of measurement separately for the three groups.

**Table 1:** Mean (SD) of age, meditation, and yoga practice (min per year), home assignments and engagement with content of group after the last session (min per week), and relative frequency of education state, yoga, and meditation practice for each group.

	Age^a^	Education^a^	Meditation practice (min/year)^a^	Yoga practice (min/year)^a^	Home assignments (min/week)^b^	Engagement since last session (min/week)^c^
Compassion and caring-based mental training (*N* = 31)	23.65 (3.23)	High School: 90.3% Bachelor: 6.5% Master: 3.2%	418.87 (1022.84) Never: 6.5% Once: 48.4% Sometimes/year: 16.1% Sometimes/month: 25.8% Daily: 3.2%	1170.48 (2279.37) Never: 6.5% Once: 29.0% Sometimes/year: 29.0% Sometimes/month: 29.0% Daily: 6.5%	32.45 (44.46)	15.00 (25.63)
Adapted MBSR training (*N* = 34)	21.94 (3.77)	High School: 97.1% Master: 2.9%	391.91 (1022.54) Never: 8.8% Once: 52.9% Sometimes/year: 11.8% Sometimes/month: 23.5% Daily: 2.9%	1559.41 (3140.73) Never: 8.8% Once: 35.3% Sometimes/year: 17.6% Sometimes/month: 23.5% Daily: 14.7%	20.15 (33.68)	17.65 (32.18)
Active control group (*N* = 26)	21.65 (2.21)	High School: 100.0%	220.96 (502.30) Never: 7.7% Once: 57.7% Sometimes/year: 15.4% Sometimes/month: 15.4% Daily: 3.8%	1550.96 (3643.67) Never: 11.5% Once: 34.6% Sometimes/year: 23.1% Sometimes/month: 19.2% Daily: 11.5%	16.54 (16.84)	7.88 (12.26)

a measured at pre-test.

b ^measured at post-test.^

c measured at follow-up.

**Table 2:** Mean (*SD*) of importance of nutrition and three highest rated reasons for a chosen vegetarian/vegan diet and relative frequency of eating habits for each group.

	Pre-test			Post-test			Follow-up		
	Eating habit	Importance nutrition^a^	Reasons for veg diet^a^^,^ ^b^	Eating habit	Importance nutrition^a^	Reasons for veg diet^a^^,^ ^b^	Eating habit	Importance nutrition^a^	Reasons for veg diet^a^^,^ ^b^
Compassion and caring-based mental training (*N* = 31)	Vegetarian/vegan: 35.5% (11) Omnivore: 64.5% (20)	4.39 (0.62)	Sustainability: 4.45 (0.93) Health: 4.18 (1.08) Moral: 4.09 (0.83)	Vegetarian/vegan: 41.9% (13) Omnivore: 58.1% (18)	4.35 (0.55)	Sustainability: 4.54 (0.66) Moral: 4.15 (0.80) Health: 3.92 (1.44)	Vegetarian/vegan: 35.5% (11) Omnivore: 64.5% (20)	4.35 (0.66)	Sustainability: 4.91 (0.30) Moral: 4.36 (0.81) Health: 4.27 (1.01)
Adapted MBSR training (*N* = 34)	Vegetarian/vegan: 44.1% (15) Omnivore: 55.9% (19)	4.38 (0.65)	Sustainability: 4.20 (1.01) Moral: 4.00 (1.20) Health: 4.00 (0.93)	Vegetarian/vegan: 50.0% (17) Omnivore: 50.0% (17)	4.35 (0.69)	Sustainability: 4.24 (0.66) Health: 4.18 (0.73) Moral: 4.12 (0.93)	Vegetarian/vegan:4.1% (15) Omnivore: 55.9% (19)	4.32 (0.73)	Sustainability: 4.33 (0.72) Moral: 4.33 (0.72) Health: 4.33 (0.72)
Active control group (*N* = 26)	Vegetarian/vegan: 30.8% (8) Omnivore: 69.2% (18)	4.08 (0.69)	Sustainability: 4.38 (0.74) Health: 4.38 (0.52) Moral: 4.25 (0.71)	Vegetarian/vegan: 42.3% (11) Omnivore: 57.7% (15)	4.12 (0.77)	Moral: 4.36 (1.03) Sustainability: 4.27 (0.79) Health: 4.09 (0.94)	Vegetarian/vegan: 42.3% (11) Omnivore: 57.7% (15)	4.27 (0.72)	Moral: 4.55 (0.69) Sustainability: 4.09 (0.83) Health: 3.73 (0.79)

a scale from 1 (“not at all”) to 5 (“very much”).

b if vegetarian or vegan was specified at “Eating habit”.

### 3.2. Effects of groups on the explicit and implicit affective attitudes toward vegetarian foods (hypothesis 1)

**Figure 2:**
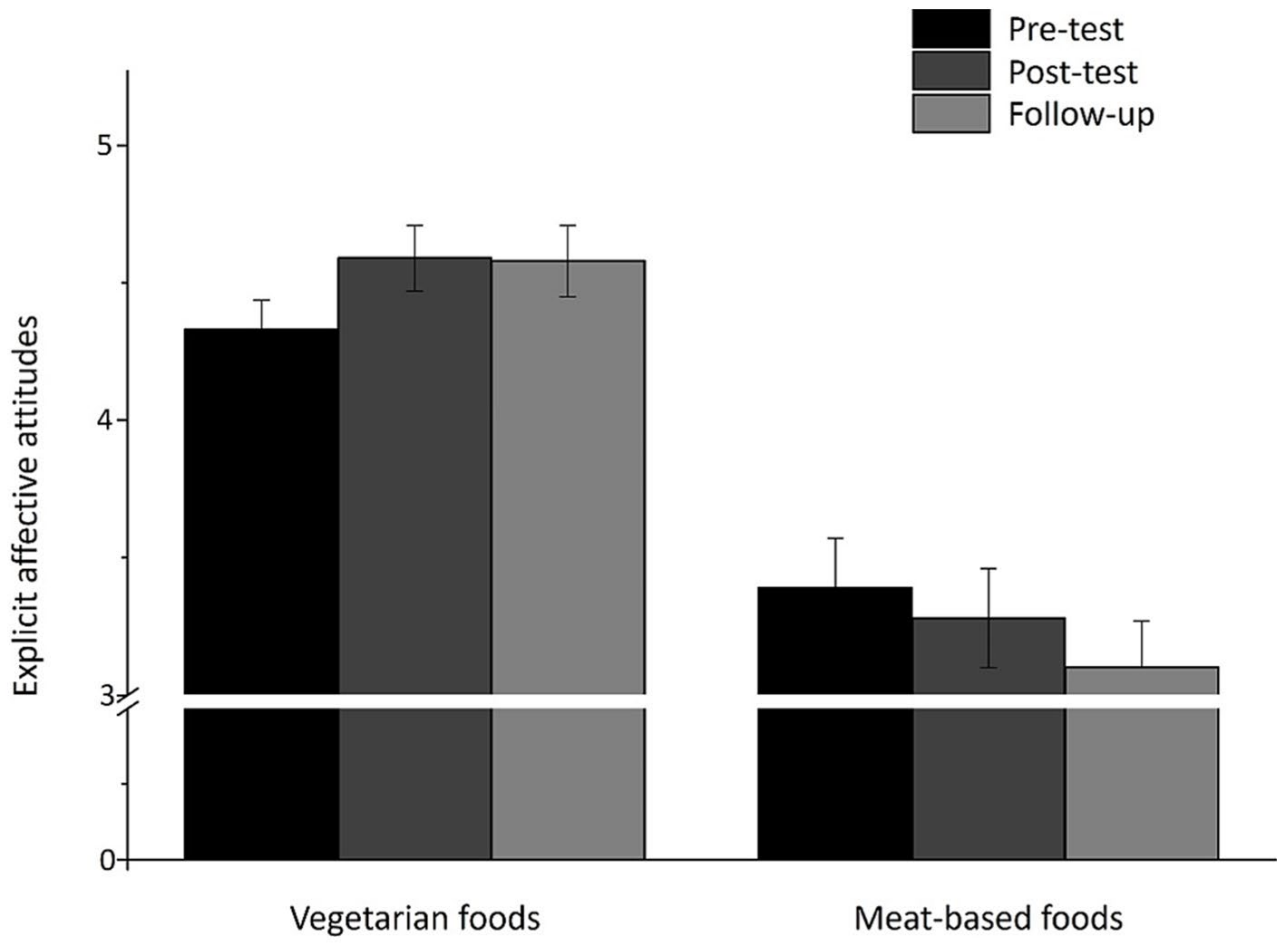
Means (SE) of the explicit affective attitudes toward vegetarian and meat-based foods in pre-test, post-test, and follow-up.

The repeated measure ANOVA for the explicit affective attitudes toward the vegetarian dishes showed a significant main effect of time, *F*(1.74, 152.82) = 4.43, *p* = 0.017, partial *η*^2^ = 0.048. There was no main effect of group (*F*(2, 88) = 0.60, *p* = 0.552), as well as no significant interaction between time and group (*F*(3.47, 152.82) = 1.21, *p* = 0.346). Subsequent performed Bonferroni-adjusted matched-pairs *t*-tests (*p* < 0.017) revealed a significant difference between the explicit attitudes in the pre-test and post-test, *t*(90) = −3.12, *p* = 0.001, 95% CI [−0.42, −0.09], *d* = 0.79, as well as pre-test and follow-up, *t*(90) = −2.20, *p* = 0.015, 95% CI [−0.46, −0.02], *d* = 1.06, but not between post-test and follow up, *t*(90) = 0.15, *p* = 0.441, see Figure 2. The repeated measure ANOVA for the implicit rating revealed no main or interaction effects.

### 3.3. Stage affiliation and explicit and implicit affective attitudes toward vegetarian foods (hypothesis 2)

In all three measurements, the explicit affective attitudes toward vegetarian foods descriptively were more positive the “higher” the stage of the participants (see Table 3). Regarding the implicit attitudes, the same tendency could be noted with one exception in the follow-up (see Table 4). The one-way ANOVA with stage affiliation as an independent variable revealed a significant difference regarding the explicit affective attitudes toward the vegetarian dishes between the different levels of stage affiliation in the pre-test, *F*(3, 87) = 4.70, *p* = 0.004, partial *η*^2^ = 0.140, the post-test, *F*(3, 87) = 5.79, *p* = 0.001, partial *η*^2^ = 166, and in the follow-up, *F*(3, 87) = 9.42, *p* < 0.001, partial *η*^2^ = 0.245. Turkey *post-hoc* analyses revealed a significant difference between pre-decision and post-action stages (pre-test: −0.97, *p* = 0.017, 95% CI [−1.81, −0.13], post-test: −1.19, *p* = 0.004, 95% CI [−2.07, −0.30], follow-up: −1.57, *p* < 0.001, 95% CI [−2.56, −0.58]) and pre-action and post-action stages (pre-test: −0.84, *p* = 0.039, 95% CI [−1.65, −0.32], post-test: −1.09, *p* = 0.040, 95% CI [−2.14, 0.55], follow-up: −1.45, *p* = 0.001, 95% CI [−2.44, −0.46]). There were no significant differences regarding implicit affective attitudes in all three measurements.

**Table 3:** Explicit affective attitudes toward vegetarian foods^a^ in the self-regulated behavioral change (SSBC).

		Pre-test			Post-test			Follow-up		
Stage	Stage affiliation	*N*	*M*	*SD*	*N*	*M*	*SD*	*N*	*M*	*SD*
Pre-decision	My meals often contain meat-based foods, and I do not intend to change that in the future.	11	3.64*^,1^	0.96	12	3.7*^,3^	1.28	10	3.4*^,5^	1.42
Pre-action	These days I’m thinking about eating vegetarian meals more often instead of meat-based ones, but I do not know exactly how to implement that yet.	12	3.77*^,2^	1.05	8	3.8*^,4^	0.83	10	3.52*^,6^	1.05
Action	I’ve decided to eat vegetarian instead of meat-based meals more often in the future, and I’ve already educated myself on how to make that happen.	15	4.35	0.84	11	4.46	0.96	9	4.38	0.85
Post-action	I prefer to eat vegetarian instead of meat-based meals as often as possible and will maintain this in the future.	53	4.61*^,1,2^	0.98	60	4.89*^,3,4^	1.07	62	4.97*^,5,6^	1.1

a scale from 1 (“not at all)” to 5 (“very much”).

^*^significant difference *p* < 0.05, superscript numbers (1–6) denote the respective significantly different means.

**Table 4:** Implicit affective attitudes toward vegetarian foods^a^ in the self-regulated behavioral change (SSBC).

		Pre-test			Post-test			Follow-up		
Stage	Stage affiliation	*N*	*M*	*SD*	*N*	*M*	*SD*	*N*	*M*	*SD*
Pre-decision	My meals often contain meat-based foods, and I do not intend to change that in the future.	11	−17.28	44.49	12	−16.7	90.44	10	−5.38	32.35
Pre-action	These days I’m thinking about eating vegetarian meals more often instead of meat-based ones, but I do not know exactly how to implement that yet.	12	24.31	56.85	8	−11.4	40.96	10	19.72	82.56
Action	I’ve decided to eat vegetarian instead of meat-based meals more often in the future, and I’ve already educated myself on how to make that happen.	15	29.26	51.15	11	8.55	30.55	9	32.08	76.88
Post-action	I prefer to eat vegetarian instead of meat-based meals as often as possible and will maintain this in the future.	53	30.55	79.44	60	20.11	57.94	62	20.98	54.4

a RT_picture-primed negative words_—RT_picture-primed positive words_; the higher the value, the more positive the attitude.

### 3.4. Effects of groups, attitudes, social and personal norms, vegetarian diet, mindfulness facets, compassion, wellbeing on the goal intention (hypothesis 3)

The results of the multiple regression analysis for goal intention in the post-test showed that 82% (adjusted *R*^2^ = 0.61) of the variance is explained, *F*(14, 76) = 11.01, *p* < 0.001, with the two significant predictors personal norm and vegetarian/vegan diet (see Table 5a). In the multiple regression analysis in the follow-up, 86% (adjusted *R*^2^ = 0.68) of the variance is explained with the model, *F*(14, 76) = 14.87, *p* < 0.001. Besides personal norms and a vegetarian/vegan diet, social norm was the third significant predictor (see Table 5b).

**Table 5a:** Regression-analysis with the criterion goal intention in the post-test.

	Goal intention (post-test)					
Variable (post-test)	*b*	*SE*	*β*	*t*	*p*	95% CI
Compassion and caring-based mental training^a^	−0.13	0.22	−0.05	−0.58	0.567	[−0.56, 0.31]
Adapted MBSR training^a^	−0.02	0.22	−0.01	−0.10	0.919	[−0.46, 0.42]
Explicit attitudes toward vegetarian food	0.09	0.09	0.08	1.00	0.320	[−0.09, 0.26]
Implicit attitudes toward vegetarian food	0.00	0.00	0.02	0.34	0.734	[0.00, 0.00]
Social norm	0.04	0.10	0.04	0.43	0.669	[−0.15, 0.23]
Personal norm	0.62	0.12	0.49	5.27	< 0.001	[0.38, 0.85]
Compassion (CS)	0.13	0.26	0.04	0.50	0.622	[−0.40, 0.66]
Wellbeing (BIT)	0.08	0.18	0.04	0.44	0.658	[−0.28, 0.44]
Nonjudging of inner experience (FFMQ)	0.17	0.12	0.13	1.43	0.157	[−0.07, 0.42]
Describing (FFMQ)	0.04	0.12	0.03	0.36	0.722	[−0.20, 0.28]
Observing (FFMQ)	0.05	0.17	0.02	0.28	0.782	[−0.29, 0.38]
Acting with awareness (FFMQ)	−0.09	0.16	−0.05	−0.56	0.576	[−0.40, 0.23]
Nonreactivity (FFMQ)	−0.03	0.15	−0.02	−0.20	0.840	[−0.33, 0.27]
Vegetarian/vegan diet	0.80	0.21	0.33	3.82	< 0.001	[0.38, 1.22]

a Reference group: Active control group.

**Table 5b:** Regression-analysis with the criterion goal intention in the follow-up.

	Goal intention (follow-up)					
Variable (follow-up)	*b*	*SE*	*β*	*t*	*p*	95% CI
Compassion and caring-based mental training^a^	−0.28	0.19	−0.11	−1.47	0.147	[−0.66, 0.10]
Adapted MBSR training^a^	−0.11	0.19	−0.05	−0.58	0.561	[−0.49, 0.27]
Explicit attitudes toward vegetarian food	0.13	0.08	0.14	1.65	0.102	[−0.03, 0.29]
Implicit attitudes toward vegetarian food	0.00	0.00	0.06	0.96	0.343	[0.00, 0.00]
Social norm	0.21	0.08	0.20	2.79	0.007	[0.06, 0.36]
Personal norm	0.57	0.11	0.43	5.06	< 0.001	[0.34, 0.79]
Compassion (CS)	0.13	0.24	0.04	0.55	0.585	[−0.34, 0.60]
Wellbeing (BIT)	0.11	0.18	0.05	0.61	0.544	[−0.24, 0.46]
Nonjudging of inner experience (FFMQ)	−0.03	0.01	−0.03	−0.35	0.731	[−0.23, 0.16]
Describing (FFMQ)	0.09	0.01	0.07	0.93	0.353	[−0.10, 0.29]
Observing (FFMQ)	−0.18	0.15	−0.09	−1.25	0.214	[−0.47, 0.11]
Acting with awareness (FFMQ)	0.19	0.15	0.11	1.28	0.205	[−0.11, 0.50]
Nonreactivity (FFMQ)	−0.09	0.13	−0.05	−0.70	0.486	[−0.36, 0.17]
Vegetarian/vegan diet	0.69	0.19	0.28	3.54	< 0.001	[0.30, 1.07]

a Reference group: Active control group.

### 3.5. Correlations between explicit and implicit affective attitudes toward vegetarian and meat-based foods (hypothesis 4)

There was a significant correlation between the explicit affective attitudes and the implicit affective attitudes toward vegetarian meals in the pre-test, *r* = 0.27, *p* = 0.011, but neither in the post-test (*r* = 0.16, *p* = 0.121) nor follow-up (*r* = −0.04, *p* = 0.719). There was no correlation between the explicit and implicit affective attitudes toward the meat dishes (pre-test: *r* = 0.06, *p* = 0.600; post-test: *r* = 0.06, *p* = 0.600; follow-up: *r* = 0.04, *p* = 0.701).

### 3.6. Difference between explicit and implicit affective attitudes toward vegetarian and meat-based foods

The paired t-test revealed a significant difference between explicit affective attitudes toward vegetarian and meat dishes in the pre-test (vegetarian: *M* = 4.34, *SD* = 1.02; meat: *M* = 3.40, *SD* = 1.79), *t*(90) = 3.90, *p* < 0.001, 95% CI [0.46, 1.42], post-test (vegetarian: *M* = 4.59, *SD* = 1.15; meat: *M* = 3.28, *SD* = 1.74), *t*(90) = 5.07, *p* < 0.001, 95% CI [0.80, 1.82], and at the time of the follow-up test (vegetarian: *M* = 4.58, *SD* = 1.26; meat: *M* = 3.11, *SD* = 1.65), *t*(90) = 5.65, *p* < 0.001, 95% CI [0.96, 1.99]. Another paired t-test also resulted in a significant difference between implicit affective attitudes toward vegetarian and meat dishes in the pre-test (vegetarian: *M* = 23.74, *SD* = 70.04; meat: *M* = −15.44, *SD* = 86.97), *t*(90) = 3.40, *p* = 0.001, 95% CI [16.28, 62.07], post-test (vegetarian: *M* = 11.09, *SD* = 60.31; meat: *M* = −4.55, *SD* = 75.61), *t*(90) = 1.70, *p* = 0.047, 95% CI [−2.69, 33.96] and at the time of the follow-up test (vegetarian: *M* = 19.04, *SD* = 58.34; meat: *M* = 1.17, *SD* = 56.38), *t*(90) = 2.42, *p* = 0.009, 95% CI [3.20, 32.53]. The explicit and implicit affective attitudes toward the pictures of the vegetarian dishes were more positive than toward the meat pictures, with a small to medium effect size for the explicit attitudes (pre-test: *d* = 0.41, post-test: *d* = 0.53, follow-up: *d* = 0.59) and small effect size for the implicit attitudes (pre-test: *d* = 0.36, post-test: *d* = 0.18, follow-up: *d* = 0.25).

### 3.7. Rating of sustainability of the vegetarian and meat foods

There was a significant difference between the personal evaluation of sustainability between the vegetarian and meat foods at the pre-test (vegetarian: *M* = 6.08, *SD* = 0.59; meat: *M* = 1.65, *SD* = 0.65), *t*(90) = 50.50, *p* < 0.001, 95% CI [4.25, 4.60], post-test (vegetarian: *M* = 6.15, *SD* = 0.53; meat: *M* = 1.55, *SD* = 0.58), *t*(90) = 60.13, *p* < 0.001, 95% CI [4.44, 4.75], and at the time of the follow-up test (vegetarian: *M* = 6.09, *SD* = 0.58; meat: *M* = 1.57, *SD* = 0.59), *t*(90) = 52.09, *p* < 0.001, 95% CI [4.35, 4.70]. This indicates a higher sustainability evaluation for the vegetarian dishes with a medium to large effect size for the three measurement time points (pre-test: *d* = 0.84, post-test: *d* = 0.73, follow-up: *d* = 0.83).

### 3.8. Exploratory analysis

In an exploratory manner, we investigated whether there were changes in mindfulness (FFMQ), compassion (CS), and wellbeing (BIT) between the pre-test and post-test, dependent on the intervention group. Regarding mindfulness, a repeated measure ANOVA showed only an effect for the factor time, *F*(1, 88) = 8.61, *p* = 0.004, partial *η*^2^ = 0.089, but not for group, *F*(2, 88) = 0.03, *p* = 0.966, partial *η*^2^ = 0.001 or the interaction between time and group, *F*(2, 88) = 1.38, *p* = 0.257, partial *η*^2^ = 0.030. The mindfulness score was higher in the post-test (*M* = 3.30, *SD* = 0.50) compared to the pre-test (*M* = 3.40, *SD* = 0.51). No significant changes in compassion and wellbeing depended on time, group, or the interaction of time and group. Furthermore, there was no correlation between the difference of the pre- and post-test in the explicit attitudes toward vegetarian food and the change in mindfulness over time, *r* = 95% CI [−0.10, 0.31]. We further investigated whether the interventions impacted the explicit affective attitudes toward meat-based foods. The repeated measure ANOVA showed a significant main effect of time, *F*(1.81, 159.40) = 5.44, *p* = 0.007, partial *η*^2^ = 0.058, but neither a main effect of group (*F*(2, 88) = 0.14, *p* = 0.870) nor a significant interaction between time and group (*F*(3.62, 159.40) = 1.19, *p* = 0.315). *Post-hoc* Bonferroni-adjusted matched-pairs *t*-tests (*p* < 0.017) revealed a significant difference between pre-test and follow-up, *t*(90) = 2.76, *p* = 0.003, 95% CI [0.08, 0.50], *d* = 1.01, as well as post-test and follow-up, *t*(90) = 2.23, *p* = 0.014, 95% CI [0.02, 0.33], *d* = 0.75 (see Figure 2).

In an additional exploratorily analysis, we also considered potential gender differences in the explicit affective attitudes. An independent sample *t*-test revealed no significant differences between women and men in their explicit affective attitudes toward vegetarian foods during the pre-test. However, there were differences at the time of post-testing with more positive attitudes in women (*M* = 4.89, *SD* = 1.10) compared to men (*M* = 4.17, *SD* = 1.09), *t*(89) = −3.06, *p = 0*.003, 95% CI [−1.18, −0.25], *d* = −0.65. Regarding explicit attitudes toward meat-based foods, the pattern was reversed, with significant gender discrepancies in both pre-test (*Z* = −4.11, *p* < 0.001, Spearman’s ρ = −0.43) and post-test (*Z* = −3.89, *p* < 0.001, Spearman’s *ρ* = −0.41), as demonstrated by a Wilcoxon-Mann–Whitney test. Men showed more positive attitudes (pre-test: *M*_Rank_ = 59.70, post-test: *M*_Rank_ = 58.96) than women (pre-test: *M*_Rank_ = 36.61, post-test: *M*_Rank_ = 37.12).

## 4. Discussion

The results showed that the explicit rating of the vegetarian foods increased significantly between pre-test and post-test, and pre-test and follow-up regardless of the assigned group. There were no significant changes in the implicit attitudes toward the vegetarian pictures. Including the SSBC, our results revealed a significant difference in the explicit affective attitudes toward vegetarian foods depending on stage affiliation between the pre-decision and post-action stages and the pre-action and post-action stages at all three testing times. Nevertheless, no such connection could be found for the implicit measurements. Also contrary to our assumptions, the multiple regression model identified only two significant predictors for goal intention in the post-test: personal norm and a vegetarian/vegan diet. In addition to these two factors, the relation between social norms and goal intention was also significant at the follow-up. Last, in contradiction to the fourth hypothesis, we found a significant correlation between the explicit and implicit affective attitudes toward vegetarian foods in the pre-test but neither in the post-test and follow-up nor between the explicit and implicit affective attitudes toward the meat-based dishes.

### 4.1. Intervention groups and explicit and implicit affective attitudes toward vegetarian foods

As stated, the intervention effect towards a more favorable rating of vegetarian foods was rather general and unrelated to the assigned intervention. Neither the adapted MBSR and the compassion and caring-based as mindfulness interventions nor the stress-reduction program of the active control group seem more suitable for improving the explicit attitudes toward vegetarian foods. A possible explanation is that these attitudinal changes are attributed to the general engagement with oneself every week for 75 min, 12 weeks long, and not specific mindfulness practice. By taking time for themselves in stressful everyday life—whether in a mindfulness course or stress-reduction training—participants reflect, connect with themselves on a deeper level, and might set inner transformations in motion. According to Woiwode et al. (2021, p. 853), “inner dimensions and transformation are essential to understand and facilitate personal and collective processes of change in terms of our awareness and relationship to ourselves, others, and the environment.” Another interesting (exploratory) result is that the interventions seem to have affected the explicit affective attitudes toward meat-based foods. However, in contrast to the attitudes toward vegetarian dishes, the rating decreased over time. Again, this effect of the intervention was independent of the assigned group. Both conceptually different mindfulness interventions and the PMR training thus might not only improve the explicit attitudes toward vegetarian foods but also worsen the attitudes toward meat dishes which is also beneficial for the choice of a sustainable diet. Our exploratory analysis showed an improvement in mindfulness measured by the FFMQ in both mindfulness intervention groups, which can be considered a control measure for the effectiveness of the curriculums. However, the mindfulness score also increased in the active control group over time. This is consistent with previous research that indicates that although mindfulness is a mechanism specific to mindfulness interventions like MBSR, stress-reduction programs such as PMR can also improve mindfulness (e.g., Agee et al., 2009; Gao et al., 2018). An explanation for this result is that there are some overlapping aspects of PMR and mindfulness curriculums like MBSR, as both programs incorporate components that cultivate attentional processes. In PMR, the participants are guided to focus attention on specific muscle groups and their contraction and relaxation. Though achieved through physical rather than mental exercise, the concentration on the present moment is quite similar to the key elements of MBSR, like awareness of the present moment and attentional control. However, the changes in the explicit attitudes and the mindfulness score did not correlate. In addition, there were no positive changes in wellbeing for both mindfulness interventions and the active control group. This null effect contradicts previous findings that mindfulness interventions affect personal wellbeing (Geiger et al., 2020) and questions wellbeing as a mechanism for the relation between mindfulness and sustainable consumption in the context of vegetarian food (Fischer et al., 2017). Also, contradictory to previous research showing that LKM may enhance compassion (Luberto et al., 2018), there was no enhancement of compassion in the compassion training group. One reason might be that the intervention time of 12 weekly sessions of 75 min was too short and not intensive enough to change wellbeing and compassion. For instance, in the study of Trautwein et al. (2020), the training modules lasted for three months and included a three days long intensive retreat, 13 weekly group sessions á 120 min, as well as daily home exercises with audio streams for guided meditations and an interface for the dyadic exercises on an internet platform and smartphone applications. Another possible explanation worth considering is that there might be a third standard set of factors related to mindfulness and sustainable behavior (Geiger et al., 2020) or sustainable nutrition in particular. For example, Wamsler et al. (2021) mention five internal transformative qualities—awareness, connections, insight, purpose, and agency—that might mediate the relationship between mindfulness and pro-environmental behavior.

### 4.2. Implementation of the SSBC

Regarding the SSBC, our results confirm the plausibility of the suggested sequence in the vegetarian context: the explicit affective attitudes were descriptively more positive the higher the stage in the model. According to Richter and Hunecke (2020), people in the first stage—the pre-decision stage—might have no problem awareness of their environmentally harmful behavior and, thus, no plan to change their diet. In the second stage, the pre-action, awareness for a necessary change is formed, but there is no concrete plan for implementation, as this concretization comes only in the action phase. In this stage, people have planned the time and realization of their new behavior. Last, the change is accomplished in the post-action stage and the new behavior has become a new habit. We found a significant difference in the explicit affective attitudes between the stages pre-decision and post-action, as well as pre-action and post-action. This link between attitudes toward vegetarian foods and an actual implemented vegetarian diet again highlights the importance of human attitudes for sustainable behavior, especially when comparing earlier stages of behavioral change and the post-action phase. This development was not reflected in the implicit attitudes, at least statistically. However, it is in line with the study of Siebertz et al. (2022), who also did not find any evidence for a possible role of implicit attitudes in the SSBC toward vegetarian and vegan nutrition. Does this mean implicit attitudes are irrelevant to the behavioral change towards a vegetarian diet? Before making such a significant conclusion, it is worth using other implicit measurements in further studies. In the SSBC, attitudes, social, and personal norms are stage-specific variables for goal intention in the pre-decision stage. Nevertheless, attitudes did not predict goal intention in our study. A reason for this might be the different ratings of explicit attitudes. In our study, participants had to explicitly rate pictures of vegetarian and vegan food. In the study of Richter and Hunecke (2020), they had to complete a questionnaire. Food pictures draw attention and activate brain areas related to reward, salience, and cognitive control (Blechert et al., 2019). This is not the case for questionnaires. There was no relationship between any of the mindfulness facets and goal intention, which contradicts previous studies that showed a relation between the mindfulness aspect of observing and goal intention (Richter and Hunecke, 2020; Siebertz et al., 2022) and describing and goal intention (Richter and Hunecke, 2020). However, this emphasizes the importance and predictive value of one’s social and personal norms. This result strengthens the normative pathway in the two-pathway model of pro-environmental behavior (Thiermann and Sheate, 2020). Nevertheless, people’s values and personal attitudes are part of the inner transformation concept. This finding again highlights the relevance of inner dimensions for sustainability (Woiwode et al., 2021).

### 4.3. Explicit and implicit affective attitudes toward vegetarian foods

We compared the explicit and implicit affective attitudes toward pictures of either vegetarian or meat-based foods. Participants rated explicitly and implicitly vegetarian dishes more positive than the foods based on meat at all three measurements before and independent of the assigned intervention group. This is in line with previous results regarding the nutrition-related sustainability of Siebertz et al. (2022). In this study, non-omnivore participants evaluated explicitly and implicitly vegetarian compared to meat-based foods more positively. The differentiation between vegetarian and omnivore could not be conducted in the study presented here due to the relatively small number of participants. The individual rating of sustainability of the shown dishes revealed that the vegetarian dishes were estimated to be more sustainable than the meat foods, suggesting that the attitudes toward vegetarian and sustainable foods could have been investigated. However, among the participants that followed a vegetarian or vegan diet, the three most reported reasons for their eating behavior choices were sustainability, morale, and health. Hopwood et al. (2020) identified health as the most common motive for non-vegetarians to consider a vegetarian diet. Further individual characteristics of vegetarians can be gender, age, education, and income, such as personality traits (Pfeiler and Egloff, 2018). Therefore, the motives for maintaining a vegetarian or vegan lifestyle are manifold and may be complex. Thus, the more positive attitude toward vegetarian dishes cannot be attributed clearly to the perceived sustainability of the shown foods. Contrary to our fourth hypothesis, there was a correlation between the explicit and implicit evaluations of vegetarian foods in the pre-test but not in the post-test and follow-up. The lack of correlation is in line with previous findings that suggested a low congruence between the explicit and implicit attitudes in the context of sustainability (Steiner et al., 2018; Jansen et al., 2021). However, we assumed there might be a congruence after the mindfulness intervention groups since mindfulness could reduce the activation of automatic associations (Lueke and Gibson, 2015). The reason for this might be the choice of the implicit measurement paradigm (see 4.4. Limitations).

### 4.4. Limitations

We implemented a controlled longitudinal intervention instead of a cross-sectional design to allow causal conclusions and interpretations of effects. However, some limitations must be considered.

First, we only considered attitudes toward vegetarian foods as one possible way of sustainable eating behavior. As stated before, there are many different types of sustainable nutrition and following a vegetarian lifestyle is just one of them. In addition, sustainable eating behavior has more dimensions than just food consumption. Factors like, for example, cultivation and production of food or recycling and disposal of packaging also must be considered as well in terms of sustainability (see Geiger et al. (2018) for an integrative cube framework of sustainable consumption behavior). Second, regarding the implementation of the SSBC, it must be noted that the sample size of each stage was rather small (e.g., 11 in pre-decision stage, see Table 3). Thus, discriminatory validity is limited. Furthermore, stage affiliation was determined by one single-choice item and was therefore only based on self-report that could be biased by other factors such as social desirability. Another major methodological drawback of our study is the lack of significant results regarding the implicit affective attitudes in our investigation. The reason might be the choice of the implicit measurement method. We used an implicit affective priming paradigm as affective motives are seen as relevant factors in environmental psychology (Steg, 2005). Another established task could be the Implicit Association test (IAT) which focuses on cognitive aspects of attitudes (see Greenwald and Lai, 2020). However, priming procedures generally suffer from lower reliability (e.g., Cameron et al., 2012). Another limitation of our investigation might be the choice of picture material in the explicit and implicit measurement. As taste varies between people, it cannot be assured that individual preferences did not impact affective attitudes. Especially in the explicit rating, the participants had to indicate whether they “like” the displayed foods regardless of sustainability aspects. As mentioned above, the reasons for a vegetarian diet are various, and no clear inferences can be derived as to whether attitudes toward sustainability were measured. In addition, as we did not monitor whether the participants were hungry or satiated during the tests, a possible sensation of hunger or appetite could also have influenced the affective evaluation of the dishes. Other limiting factors underlie the structure of our sample. Demographic analyses of the three intervention groups revealed significant discrepancies regarding age and attended group sessions. Moreover, our sample included both vegetarian/vegan and omnivorous participants. Previous research indicated that there are attitude differences between vegetarians/vegans and omnivores in terms of a more positive attitude toward vegetarian products in vegetarians compared to omnivores and a more positive attitude toward meat in omnivores compared to vegetarians (e.g., Barnes-Holmes et al., 2010; Siebertz et al., 2022). Thus, it might be more crucial to improve the attitudes toward vegetarian foods especially of omnivorous people. Future studies might profit from rather omnivorous samples to determine the impact of mindfulness interventions on the attitudes toward vegetarian foods, as larger effects can be achieved in this population. Last, as our exploratory analysis revealed gender differences in the explicit affective attitudes toward both vegetarian and meat-based foods. This discrepancy is in line with previous research suggesting that nutritional attitudes and eating habits might vary with gender (e.g., Love and Sulikowski, 2018), implying that gender should be taken into account as a factor in future studies on intervention effects on attitudes toward foods.

### 4.5. Implications for research and practice

In our intervention study, there was an improvement in the explicit affective attitudes toward vegetarian foods for all three 12 weeks long curriculums—compassion and caring-based intervention with LKM, a rather attention-focused adapted MBSR course, and as an active control group PMR training—despite the group assignment. These findings highlight the value of implementing mindfulness and stress-reduction trainings as potential interventions to promote vegetarianism and, thus, a way of sustainable food consumption. Accordingly, more courses of this form should be made accessible to a broad public as possible. However, implementing the SSBC revealed social and personal norms as significant predictors of goal intention, thus aiming for a vegetarian diet. Future studies should focus more on social and personal norms and values as changes in individual inner dimensions are promising for sustainability and possibly in particular sustainable nutrition. Last, since our study showed no significant results for the implicit aspects of attitudes, researchers should consider different implicit measurements to validate their results and figure out the most appropriate method in the specific case.

## Data availability statement

The datasets presented in this study can be found in online repositories. The names of the repository/repositories and accession number(s) can be found at: https://osf.io/x9jaq/?view_only=9a68fdb44e6f4b1d986565492dda9202.

## Ethics statement

The studies involving humans were approved by the ethics board of the University of Regensburg (Reference number: 20-1740-101). The studies were conducted in accordance with the local legislation and institutional requirements. The participants provided their written informed consent to participate in this study.

## Author contributions

PJ designed the study and developed the theoretical framework. AW conducted the implementation, organized the project, and wrote the first draft of the manuscript. All authors performed the statistical analyses, edited the manuscript, read, and approved the final submitted version.

## Funding

This research was financially supported by EDEN foundation (Im Stifterverband für die Deutsche Wissenschaft; Barkhovenallee 1 45239 Essen), grand ID S0289/10048/20. There was no involvement in study design, collection, analysis, and interpretation of data, or writing of the manuscript.

## Conflict of interest

The authors declare that the research was conducted in the absence of any commercial or financial relationships that could be construed as a potential conflict of interest.

## Publisher’s note

All claims expressed in this article are solely those of the authors and do not necessarily represent those of their affiliated organizations, or those of the publisher, the editors and the reviewers. Any product that may be evaluated in this article, or claim that may be made by its manufacturer, is not guaranteed or endorsed by the publisher.

## Footnotes

^1^https://osf.io/gbvua
